# Supplementary material for: Physiological stress in response to multitasking and work interruptions: Study protocol
Source: PLoS One. 2022 Feb 8;17(2):e0263785. doi: 10.1371/journal.pone.0263785 (PMC8824354; doi:10.1371/journal.pone.0263785)
Supplement: S1 File — Questionnaire that will be used for the assessment of sample characteristics. In the actual study, a German version will be used. (PDF) [file pone.0263785.s001.pdf]

S1: Questionnaire for the assessment of sample characteristics (Becker et al.)

Age (in years): __________

Sex

- Female
- Male
- Other
- Do not want to answer this question

If female,

- Do you have a monthly cycle?

  If yes,
  - Is the cycle regular?
  - How long is a menstrual cycle on average (in days)?
  - When was the first day of the last menstruation?

  If no,
  - Why are you not having a regular cycle?
    - Menopause
    - Pregnancy
    - Hysterectomy (surgical removal of the uterus)
    - Use of contraceptives
    - Other
    - I do not know
    - Do not want to answer

Ethnicity

- White
- African American
- Arabic
- Asian
- Other: ________
- Do not want to answer

Marital status

- Single
- Married
- Relationship
- Living separately
- Divorced
- Widowed

Occupation

- Unemployed
- Apprentice
- Student
- Housewife

- Employee
- Self-employed
- Official
- Parental leave
- Sabbatical
- Retired

Highest educational degree

- No educational degree
- Certificate of secondary education
- Secondary school level
- Vocational diploma
- General qualification for university entrance
- Bachelor's degree
- Diploma or master's degree
- Ph.D.
- Habilitation

Is German your mother tongue?

- Yes
- No

Are you currently enrolled as a student?

- Yes
  If yes, what is your subject? ________
- No

Height (in cm): __________

Weight (in kg): __________

Smoking status

Do you smoke?

- Yes (more than 5 cigarettes a week)
- Sometimes (less than 5 cigarettes a week)
- Non-smoker

Diseases (physical and mental health/illness)

Are you currently suffering from one of the following diseases?

- Diseases of the nervous system (e.g., dementia, epilepsy, cerebral haemorrhage, brain tumor, migraine, Parkinson's disease, multiple sclerosis)
- Eye disorders (e.g., glaucoma, cataracts)
- Acute disease symptoms and inflammation (e.g., asthma symptoms, cystitis, allergic coryza, sinusitis, flu, laryngitis, tonsillitis, otitis media, bronchitis, appendicitis, urinary tract infection, pneumonia)
- Chronic ear diseases (e.g., tinnitus, hearing loss)

- Diseases of the cardiovascular system (e.g., atherosclerosis, high blood pressure, blood clotting disorders, heart failure, heart attack, stroke, thrombosis)
- Digestive system disorders (e.g., gastric bleeding, gastric ulcer, inflammation of the gastric mucosa, Crohn's disease, esophagitis)
- Diseases of the liver, gall bladder and pancreas (e.g., fatty liver, gallbladder inflammation, gallstones, jaundice, hepatitis, cirrhosis of the liver, inflammation of the pancreas)
- Diseases of the skeleton, muscles, and connective tissue (e.g., arthritis, osteoarthritis, herniated disc, gout, seizures, osteoporosis, rheumatism, back pain)
- Skin complaints (e.g., acne, neurodermatitis, psoriasis)
- Metabolic diseases (e.g., Addinson’s syndrome, Cushing’s syndrome, diabetes mellitus, hyperthyroidism or hypothyroidism)
- Diseases of the kidneys, urinary tract, and genital organs (e.g., bladder stones, urinary incontinence, kidney failure, kidney stones)
- Blood disorders (e.g., iron deficiency, leukemia)
- Infectious and parasitic diseases (e.g., borreliosis, cholera, yellow fever, hepatitis, HIV/AIDS, influenza, malaria, measles, rubella, syphilis, tuberculosis, chickenpox)
- Cancer (e.g., pancreatic cancer, bladder cancer, breast cancer, colon cancer, cervical cancer, skin cancer, testicular cancer, lung cancer, kidney cancer, stomach cancer, prostate cancer, vocal cord cancer)
- Obesity (BMI > 35 kg/m^2)
- No diseases

Medication

Please fill-in your regular medication intake (dose, frequency, reason for; prescription and non-prescription)

____

____

____
