# Supplementary material for: Physiological stress in response to multitasking and work interruptions: Study protocol
Source: PLoS One. 2022 Feb 8;17(2):e0263785. doi: 10.1371/journal.pone.0263785 (PMC8824354; doi:10.1371/journal.pone.0263785)
Supplement: S2 File — Visual-analogous scales with which perceived stress, tiredness, and exertion will be assessed. In the actual study, a German version will be used. (PDF) [file pone.0263785.s002.pdf]

S2: Assessment of perceived stress, tiredness, and effort (Becker et al.)

How **stressed** do you feel **currently**?

Not stressed at all — **Extremely stressed**

1	2	3	4	5	6	7	8	9	10
☐	☐	☐	☐	☐	☐	☐	☐	☐	☐

How **tired** do you feel **currently**?

Not tired at all — Extremely **tired**

1	2	3	4	5	6	7	8	9	10
☐	☐	☐	☐	☐	☐	☐	☐	☐	☐

How **exerted** do you feel **currently**?

Not exerted at all — Extremely **exerted**

1	2	3	4	5	6	7	8	9	10
☐	☐	☐	☐	☐	☐	☐	☐	☐	☐
