# Supplementary material for: Physiological stress in response to multitasking and work interruptions: Study protocol
Source: PLoS One. 2022 Feb 8;17(2):e0263785. doi: 10.1371/journal.pone.0263785 (PMC8824354; doi:10.1371/journal.pone.0263785)
Supplement: S3 File — Questionnaire that will be used for the assessment of multitasking. In the actual study, a German version will be used. (PDF) [file pone.0263785.s003.pdf]

S3: Questionnaire for the assessment of multitasking (Becker et al.)

1. What does the term multitasking mean for you?

____

____

____

2. Please give examples for multitasking tasks.

____

____

____

3. To what extent do the following statements apply to you in general?
On most days…

	Not correct at all (1)	Rather does not apply (2)	Depends (3)	Rather applies (4)	That is completely right (5)
… I am busy with several things at the same time.					
… I work on more than one task at the same time by switching back and forth between tasks within a short period of time.					
… I do not devote myself to a new task until I have finished with the current one.					
… I break off a task several times to devote myself to other tasks.					

… I am expected to do multiple tasks at the same time.					
… due to the high time pressure, I am forced to work on tasks in parallel instead of one after the other.					
… I need to focus my attention on different tasks or situations at the same time.					

If '… … I break off a task several times to devote myself to other tasks' == '4' or '5',

4. What are the most common causes for interrupting your tasks?

- Disturbances
- New tasks
- Variety
- Time pressure
- Other: __________
